# Evolution of Bystander Intention to Perform Resuscitation Since Last Training: Web-Based Survey

**DOI:** 10.2196/24798

**Published:** 2020-11-30

**Authors:** Simon Regard, Django Rosa, Mélanie Suppan, Chiara Giangaspero, Robert Larribau, Marc Niquille, François Sarasin, Laurent Suppan

**Affiliations:** 1 Division of Emergency Medicine Department of Anesthesiology, Clinical Pharmacology, Intensive Care and Emergency Medicine University of Geneva Hospitals and Faculty of Medicine Geneva Switzerland; 2 Division of Anesthesiology Department of Anesthesiology, Clinical Pharmacology, Intensive Care and Emergency Medicine University of Geneva Hospitals and Faculty of Medicine Geneva Switzerland

**Keywords:** out-of-hospital cardiac arrest, cardiopulmonary resuscitation, basic life support, confidence, first aid, bystander, behavior, cardiac arrest, heart attack, intention, resuscitation, survey, attitude, belief

## Abstract

**Background:**

Victims of out-of-hospital cardiac arrest (OHCA) have higher survival rates and more favorable neurological outcomes when basic life support (BLS) maneuvers are initiated quickly after collapse. Although more than half of OHCAs are witnessed, BLS is infrequently provided, thereby worsening the survival and neurological prognoses of OHCA victims. According to the theory of planned behavior, the probability of executing an action is strongly linked to the intention of performing it. This intention is determined by three distinct dimensions: attitude, subjective normative beliefs, and control beliefs. We hypothesized that there could be a decrease in one or more of these dimensions even shortly after the last BLS training session.

**Objective:**

The aim of this study was to measure the variation of the three dimensions of the intention to perform resuscitation according to the time elapsed since the last first-aid course.

**Methods:**

Between January and April 2019, the two largest companies delivering first-aid courses in the region of Geneva, Switzerland sent invitation emails on our behalf to people who had followed a first-aid course between January 2014 and December 2018. Participants were asked to answer a set of 17 psychometric questions based on a 4-point Likert scale (“I don’t agree,” “I partially agree,” “I agree,” and “I totally agree”) designed to assess the three dimensions of the intention to perform resuscitation. The primary outcome was the difference in each of these dimensions between participants who had followed a first-aid course less than 6 months before taking the questionnaire and those who took the questionnaire more than 6 months and up to 5 years after following such a course. Secondary outcomes were the change in each dimension using cutoffs at 1 year and 2 years, and the change regarding each individual question using cutoffs at 6 months, 1 year, and 2 years. Univariate and multivariable linear regression were used for analyses.

**Results:**

A total of 204 surveys (76%) were analyzed. After adjustment, control beliefs was the only dimension that was significantly lower in participants who took the questionnaire more than 6 months after their last BLS course (*P*<.001). Resisting diffusion of responsibility, a key element of subjective normative beliefs, was also less likely in this group (*P*=.001). By contrast, members of this group were less afraid of disease transmission (*P*=.03). However, fear of legal action was higher in this group (*P*=.02).

**Conclusions:**

Control beliefs already show a significant decrease 6 months after the last first-aid course. Short interventions should be designed to restore this dimension to its immediate postcourse state. This could enhance the provision of BLS maneuvers in cases of OHCA.

## Introduction

Survival after out-of-hospital cardiac arrest (OHCA) is estimated at around 10% in Europe, with recent studies showing marked differences between regions [1]. Bystander-initiated cardiopulmonary resuscitation (CPR) is one of the most important factors explaining these differences and has been shown to increase the survival rate by three times [1-3]. Immediate CPR initiation after collapse is particularly important, as there is a 10% decrease in survival rate for every minute spent without CPR [4]. In spite of its significant benefits, and although more than half of OHCAs are witnessed, CPR is only provided for less than half of victims, with rates varying widely from 19.1% to 79.0% [5]. As ambulances rarely arrive on scene before 10 minutes, the probability of survival is limited unless CPR has been initiated before the arrival of professional rescuers [4,6]. The deployment of comprehensive OHCA management programs has been shown to improve both survival and neurological outcomes [7], and relies heavily on the training of lay people who will be and feel able to initiate CPR quickly after collapse.

According to the theory of planned behavior, the probability of executing an action is strongly linked to the intention of performing it [8,9]. Intention is determined by three distinct dimensions [10]. The first, attitude, relates to the bystander’s beliefs. Regarding resuscitation, a positive attitude would be to think that performing CPR could save a life. The second dimension, subjective normative beliefs, is the person’s perception of the judgment close relatives might have regarding one’s actions. In the context of first aid, this would be a consideration that one’s friends would approve, or even be proud, if one performed resuscitation. The third and last dimension, control beliefs, is the confidence in one’s own ability to perform resuscitation [8,9].

In Switzerland, the rates of successful OHCA resuscitation vary from 10% to 17% [1,11]. These rates are not as high as could be expected given the rather high proportion of people having followed a CPR training course, which is mandatory to obtain a driving license in this country. This could be explained, at least in part, by the fact that basic life support (BLS) course participants lose both skills and confidence in their ability to perform CPR within a matter of weeks after completing a CPR training course [12-14]. We hypothesized that the intention to perform CPR might also decrease over time, thereby further decreasing the rate of bystander-initiated resuscitation and consequently of successful OHCA resuscitations.

The aim of this study was to measure the variation of the three dimensions of the intention to perform CPR according to the time elapsed since the last first-aid course. Identification of any significant difference could potentially help to design specific interventions and hopefully improve the rate of bystander-initiated resuscitation.

## Methods

### Design

This was a closed web-based questionnaire study following the CHERRIES [15] guidelines, which was conducted between January and April 2019. The regional ethics committee issued a nonobjection statement (ID 2018-01382) as such surveys do not fall within the scope of the Swiss federal act on research involving human beings [16].

### Participants

The two largest companies providing first-aid courses in Geneva (Association Genevoise des Sections de Samaritains, a Red Cross–affiliated national society, and Firstmed, a privately owned company) were asked for a list of email addresses of former CPR course participants. To protect individual data, both societies refused to send us such a list directly but agreed to dispatch emails on behalf of the investigators. They were therefore provided with a generic text containing summarized information about the study along with the link to the online survey. Both companies were asked to send this email to participants who had followed a first-aid course between January 2014 and December 2018.

Emails were sent between January and April 2019. Although the exact number of sent and “bouncing” emails had been asked for, these data could not be gathered as one of the two companies experienced technical problems with their mailing system. Reminders could not be sent as per the request of both companies.

No financial incentive was given to participate in this study.

### Survey

A website based on the Joomla! 3.9 content management system (Open Source Matters) was specifically designed for this study. The Community Surveys 5 component (CoreJoomla) was used to create the online survey and record the answers in an encrypted MariaDB 5.5.5 database (MariaDB Corporation AB) located on a Swiss server. As this was a closed study, and to ensure irreversible anonymization, we decided not to use either cookies or internet protocol address restrictions. A log search was nevertheless performed to identify potential duplicate entries.

The survey itself was displayed over 4 pages. The first 2 pages were designed to gather demographic data. The 17 questions were displayed over pages 3 and 4, which contained 9 and 8 questions, respectively. The system ensured that participants had answered all of the questions on a page before allowing them to move forward. All answers could be reviewed and changed as long as the survey was not finalized.

Upon loading, the website immediately displayed a summarized consent form and a confidentiality notice as well as a link to a detailed description of the study. A statement regarding data collection and storage was also shown, and the purpose and duration of the survey were detailed. Participants were informed that they could decide to leave the study at any time, and were given an email address they could use to contact the investigators. No personal data were collected.

A first set of general questions was created to gather demographic data, determine the time elapsed since the last CPR training course, and record information regarding the number of prior CPR courses followed. A set of 17 psychometric questions was then designed to assess each dimension of the intention to perform CPR (Multimedia Appendix 1). Ten questions were adapted from the Canadian national survey performed by Vaillancourt et al [8] in 2013. Seven more questions were created to assess specific factors that could further affect the dimensions of the intention to perform CPR and might therefore prevent bystanders from starting CPR. Among these latter factors, usually referred to as “barriers,” fear of disease, fear of incorrectly performing CPR, and fear of hurting the victim were evaluated [17-19]. Answers to all psychometric questions were based on a 4-point Likert scale (“I don’t agree,” “I partially agree,” “I agree,” and “I totally agree”).

The survey and the data extraction mechanism were thoroughly tested by all investigators prior to the launch of the study.

### Measures

The primary outcome was the difference in each of the three dimensions of the intention to perform resuscitation between participants who had followed a first-aid course less than 6 months before taking the questionnaire and those who took the questionnaire more than 6 months and up to 5 years after following such a course. The investigators decided to use a 6-month cutoff since the alternative of asking lay people to attend an on-site refresher course so soon after the last training course would be unlikely. Nevertheless, offering a short, targeted, and portable intervention after this time span might be considered.

Secondary outcomes were the changes in each of the three dimensions using a 1-year and then a 2-year cutoff rather than the 6-month cutoff used to compute the primary outcome, and the change in each individual question using the 6-month, 1-year, and 2-year cutoffs.

### Analyses

Survey data were extracted to a comma-separated value file and imported in Stata 16.0 (Stata Corp LLC). Records were searched for potential duplicate entries as per our protocol. Health care professionals, students of health care professions, and participants who had not followed a CPR course during the previous 5 years were excluded. Incomplete surveys were also excluded.

Stata was used for statistical analysis. Numerical values (–1, –0.5, 0.5, and 1) were attributed to each of the 4 answers gathered through the use of Likert scales, with positive values assigned to the answers that were in favor of the intention to perform resuscitation. All survey questions carried the same weight and were summed by dimension. Univariate linear regression was used to assess the variation of each specific dimension of the intention to perform resuscitation according to the time elapsed since the last first aid course. A multivariable linear regression model was used to identify the effect training centers or age groups might have had on these variations. A double-sided *P* value <.05 was considered significant.

The original dataset is available in the Mendeley Data repository [20].

## Results

**Figure 1:**
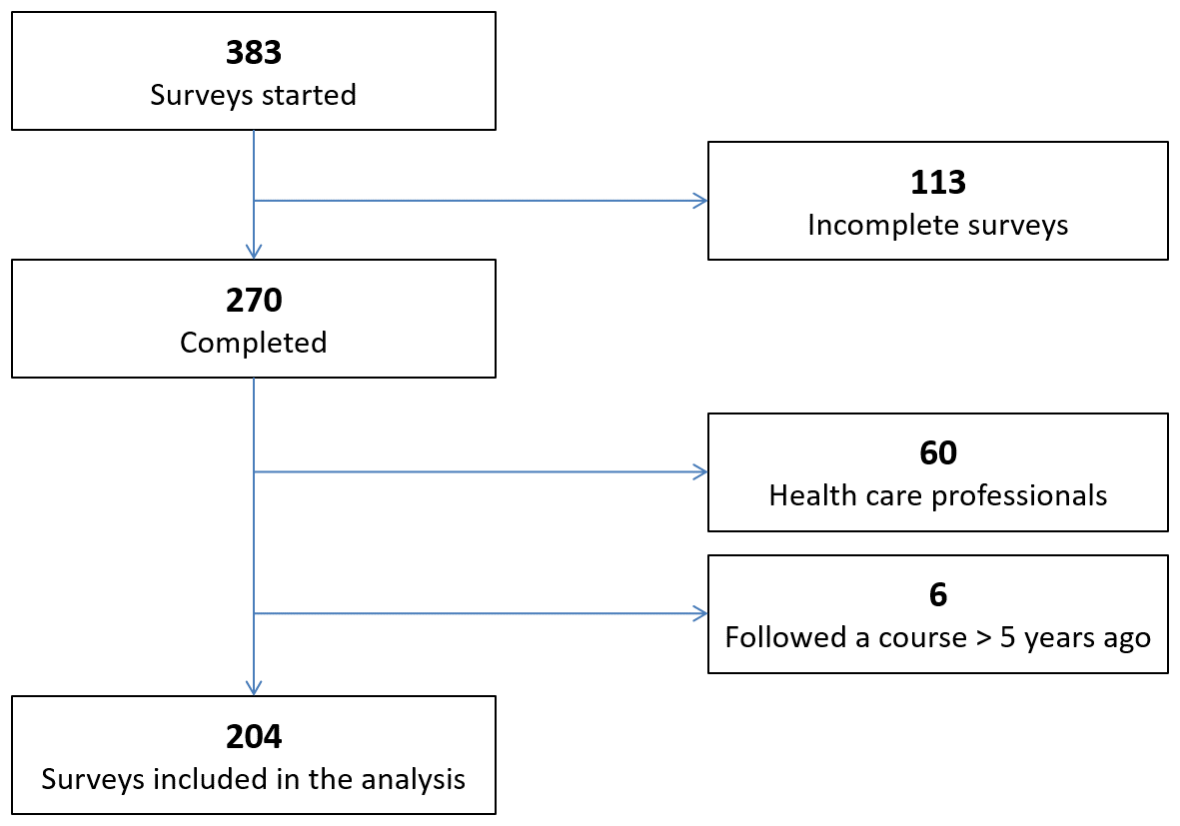
Flowchart of the inclusion of former first-aid course participants (Geneva, Switzerland, 2019).

Overall, 383 surveys were started, 270 (70.5%) of which were completed. A total of 204 of the completed surveys (75.6%) were analyzed after application of the exclusion criteria (Figure 1). No data suggestive of duplicate entry was identified.

Characteristics of the participants, including the number of prior BLS courses, are described in Table 1.

Participants who took the questionnaire more than 6 months after their last first-aid course had significantly lower scores regarding control beliefs and subjective normative beliefs (Table 2). The difference was particularly important regarding control beliefs, with 4 out of 5 questions displaying significant differences. Regarding subjective normative beliefs, diffusion of responsibility was the only element to be significantly lower in the group of participants who had taken the questionnaire more than 6 months after the last course. After adjusting for age group and training center, there was no change in either the direction of the effect or of its magnitude regarding control beliefs (*P*<.001), whereas the difference regarding subjective normative beliefs did not change direction but failed to achieve significance after adjustment (*P*=.06). The direction and the amplitude of the diffusion of responsibility element remained unchanged after adjustment (*P*<.001).

**Table 1:** Characteristics of the former first-aid course participants included in the analysis (N=204).^a^

Characteristic		Last course followed ≤6 months before (n=85)	Last course followed >6 months before (n=119)	*P* value^b^
**Education, n (%)**				.045
	Mandatory education	3 (4)	3 (2.5)	
	Professional diploma	18 (21)	34 (28.6)	
	Secondary education	33 (39)	22 (18.5)	
	High school	8 (9)	15 (12.6)	
	University	21 (25)	39 (32.8)	
	Other	2 (2)	6 (5.0)	
**Marital status, n (%)**				.74
	Single	48 (56)	63 (52.9)	
	In a relationship	16 (19)	29 (24.4)	
	Married	21 (25)	26 (21.8)	
	Widowed	0 (0)	1 (0.8)	
**Age category (years), n (** *%* **)**				<.001
	<18	18 (21)	3 (2.5)	
	18-25	32 (38)	46 (38.7)	
	26-30	6 (7)	10 (8.4)	
	31-35	5 (6)	11 (9.2)	
	36-40	8 (9)	3 (2.5)	
	41-45	1 (1)	8 (6.7)	
	46-50	6 (7)	15 (12.6)	
	51-55	6 (7)	17 (14.3)	
	>55	3 (4)	6 (5.0)	
**Sex, n (%)**				.09
	Female	66 (78)	79 (66.4)	
	Male	19 (22)	40 (33.6)	
**Number of BLS^c^courses followed, n (%)**				.29
	1	42 (49)	59 (49.6)	
	2	15 (18)	32 (26.9)	
	3	13 (15)	11 (9.2)	
	≥4	15 (18)	17 (14.3)	
**Training center**				<.001
	AGSS^d^	43 (51)	107 (89.9)	
	Firstmed	35 (41)	2 (1.7)	
	Other	7 (8)	10 (8.4)	

^a^Totals may not equal 100% due to rounding.

^b^*P*-values were calculated using the Fisher exact test.

^c^BLS: basic life support.

^d^AGSS: Association Genevoise des Sections de Samaritains.

**Table 2:** Univariate analysis of the three dimensions of the intention to perform resuscitation in first-aid course participants (N=204).

Dimension and questions^a^		Last course followed ≤6 months before, mean (95% CI)		Last course followed >6 months before, mean (95% CI)		*P* value	
**Attitude**		6.06 (5.68-6.45)		6.06 (5.79-6.33)		.99	
	Thinking that performing resuscitation could save a life		0.78 (0.70-0.87)		0.80 (0.74-0.87)		.79
	Knowing the importance of starting a resuscitation before EMS^b^ arrival		0.91 (0.84-0.98)		0.92 (0.86-0.97)		.93
	Not being afraid of disease transmission		0.47 (0.33-0.62)		0.66 (0.55-0.77)		.03
	Not being afraid of hurting the victim by performing CPR^c^		0.91 (0.85-0.98)		0.84 (0.77-0.91)		.14
	Not being afraid of worsening the victim’s condition		0.69 (0.57-0.82)		0.75 (0.68-0.83)		.41
	Not being afraid of legal action		0.78 (0.67-0.88)		0.59 (0.48-0.70)		.02
	Being proud of performing resuscitation successfully		0.61 (0.48-0.74)		0.58 (0.47-0.69)		.76
	Belief that knowing CPR is important for society		0.91 (0.85-0.96)		0.92 (0.89-0.96)		.59
**Subjective normative beliefs**		1.90 (1.61-2.19)		1.45 (1.18-1.73)		.03	
	Belief that relatives would be proud if the participant performed resuscitation		0.54 (0.42-0.67)		0.57 (0.46-0.68)		.72
	Belief that relatives want the subject to resuscitate them if needed		0.56 (0.43-0.70)		0.45 (0.32-0.57)		.19
	Knowing that relatives are the most likely victim		0.02 (–0.13-0.18)		–0.6 (–0.19-0.07)		.42
	Diffusion of responsibility		0.77 (0.68-0.86)		0.50 (0.38-0.62)		.001
**Control beliefs**		3.62 (3.31-3.93)		2.18 (1.83-2.53)		<.001	
	Knowledge of the emergency number		0.91 (0.85-0.96)		0.70 (0.59-0.81)		.004
	Feeling able to resuscitate		0.56 (0.45-0.67)		0.16 (0.04-0.29)		<.001
	Feeling able to recognize a cardiac arrest		0.68 (0.58-0.77)		0.28 (0.17-0.39)		<.001
	Not believing that only health care professionals can adequately perform resuscitation		0.77 (0.67-0.87)		0.72 (0.64-0.81)		.46
	Knowing how to perform a resuscitation		0.70 (0.61-0.79)		0.31 (0.20-0.41)		<.001

^a^For individual questions, scores can range from –1.0 to +1.0; a positive score indicates an answer in favor of the intention to perform resuscitation.

^b^EMS: emergency medical services.

^c^CPR: cardiopulmonary resuscitation.

The association between the time elapsed since the last BLS course and the fear of catching a disease while providing CPR disappeared after adjustment (*P*=.23) when the cutoff was set at 6 months. However, this association was significant (after adjustment) when the cutoff was set at 1 year (*P*=.02) (Multimedia Appendix 2) and even more so when the cutoff was set at 2 years (*P*=.01) (Multimedia Appendix 3). The direction of the effect did not change: participants who had followed a first-aid course more recently were more afraid of catching a disease in all analyses.

Fear of legal action was higher in participants who took the survey more than 6 months after having followed their last BLS course. The direction and the amplitude of this association did not change after adjustment (*P*=.02).

## Discussion

### Principal Findings

Control beliefs, including knowledge of the emergency number to dial in case of cardiac arrest, already showed a significant decrease only 6 months after the last BLS course. Although some authors have advocated for a much shorter period than the recommended 2-year interval between BLS refresher courses given the need to freshen up CPR skills [21,22], the results of this study show that the intention to perform resuscitation also needs to be restored or at least preserved. Nevertheless, other authors have emphasized that aiming for refresher courses at more frequent intervals was likely unrealistic as even highly motivated lay rescuers would lack either time or money [23,24], and having to perform retraining sessions too frequently might lead to disinterest [25]. Other means must therefore be sought to allow for frequent yet short refresher interventions [26-28]. Such interventions should target critical elements such as diffusion of responsibility [29], which quickly rises after a BLS course and might lead to delays before initiation of CPR, thereby increasing the no-flow time and worsening the patient’s prognosis [14]. In the context of the current COVID-19 pandemic, distance interventions, whether asynchronous or synchronous, have been developed rapidly and many have met with success [30,31]. Interactivity has been shown to increase engagement, and can be achieved through the creation of eLearning modules or of serious games for asynchronous interventions [32], or by the organization of webinars when synchronous interventions are deemed preferable [33].

The participants who took the questionnaire less than 6 months after following their last BLS course were significantly younger and less likely to have been trained by the Red Cross–affiliated center. Although adjusting for these variables nullified the statistical significance initially found regarding subjective normative beliefs when the cutoff was set at 6 months, the change regarding this dimension was still significant after adjustment when the cutoff was set at 2 years. This effect was mostly related to the diffusion of responsibility element. Victims of the so-called bystander effect (ie, being less likely to help a victim when other people are present [34]) may be more prone to act if they feel confident and qualified. Thus, short interventions showcasing realistic examples of diffusion of responsibility are by themselves fighting against the phenomenon and encouraging action. Recently, a scoping review conducted as part of the update process of the international consensus on CPR and emergency cardiovascular care science with treatment recommendations concluded that specific community initiatives and bundles of educational interventions could help improve the rate of bystander-initiated CPR [35]. It has also been shown that diffusion of responsibility depends on the level of danger the victim faces [34]. There could also be a significant and lasting effect of starting CPR training at an earlier stage than currently practiced in Switzerland. Many studies have indeed provided evidence that BLS training yields excellent results in school-aged children [36,37] in whom BLS maneuvers can be taught in less time and with better results [38]. Providing junior medical students with BLS courses early in their curriculum could also prove beneficial as they are expected to take action in case of an emergency [39,40] although their CPR knowledge and skills are generally limited [41-43]. Recently, an initiative including the use of asynchronous distance learning has emerged to promote the inclusion of junior medical students in first-responder systems [44].

Although Vaillancourt et al [8] used the same theoretical model in their 2013 survey, we refrained from using the exact same question set. We considered the theory of planned behavior model to be perfectly valid; however, the way questions are phrased influences the answers given by the participants, their understanding of the problematic, and their willingness to complete the survey [45]. Moreover, some questions were added to address specific issues that were not taken into account in the original survey. For example, subjective normative beliefs were further assessed by asking whether relatives would be proud if one performed resuscitation. Questions related to control beliefs were further assessed by asking whether the participant thought that that only health care professionals would be able to correctly perform CPR. Four other questions were asked regarding attitude, including the participant’s take on the impact of resuscitation on society, fear of doing more harm, and fear of catching a disease [14].

Strangely enough, although the survey was conducted before the COVID-19 pandemic, participants who had followed a BLS course in the year preceding the survey were more afraid of disease transmission. As this study was not designed to investigate this unexpected finding, its cause is not easily determined but could be a difference in course contents. A change in the guidelines could hardly play a role in this result, as the study took place in 2019 with the last major guidelines issued in 2015 [46]. Although actions could be taken to mitigate this fear, their timeliness must be assessed with regard to the current COVID-19 pandemic [47].

Of particular concern is the fact that more than half of the participants were unaware that the probability of performing CPR was higher on a relative than on a stranger. Whether this item belongs to control beliefs as suggested by Vaillancourt et al [8] or to subjective normal beliefs as suggested by the results of this study can long be debated, but the critical importance of emphasizing and spreading this message is undeniable. Indeed, lay rescuers who performed CPR for OHCA have described subsequent emotional and social difficulties [48], which may be amplified when CPR has to be performed on a close relation rather than on a stranger. Helping lay rescuers recognize this fact might help better prepare them and can ultimately avoid some of the negative psychological consequences [48,49]. Moreover, knowledge that friends and family might be efficiently helped by provision of BLS maneuvers might increase the motivation of lay rescuers in acquiring and maintaining such critical skills [50].

On a more positive note, the attitude and subjective normative beliefs dimensions were globally preserved even 2 years after the last BLS course. The fact that health care professionals are not the only people able to correctly perform CPR now seems to be well-recognized [51]. However, fear of litigation seems to increase with time, and specific reminders of local or regional legislation should be undertaken. In Switzerland, the federal law clearly states that one should help in case of emergency, but that no legal consequence can ensue should the rescuer fail [52].

Although this study has some strengths such as the relatively high number of participants despite the absence of mail reminders and the absence of outcome assessment bias thanks to electronic data recording, some limitations must be acknowledged. Lack of email reminders might have led to selection bias, as the proportion of highly motivated participants might be higher in this setting. Indeed, the high proportion of participants who had followed 2 or more BLS courses is potentially concerning, particularly given the low rate of bystander-initiated CPR in the literature and the obligation of following a BLS course to obtain a driving license in Switzerland. Nevertheless, this might, if anything, have dampened the effect of the time elapsed since the last BLS course, and led to underestimation rather than overestimation. Another limitation is that, given the aforementioned technical issues, the actual participation rate could not be calculated as the number of sent and bouncing emails could not be obtained. Furthermore, we were unable to ascertain the actual number of first-aid course participants during the study period as both companies were either reluctant or unable to provide us with these figures. Had we been able to obtain these data, they would still have been questionable. Indeed, even though we specifically asked both training companies to send invitation emails only to participants who had completed a first-aid course in the last 5 years, 6 participants were excluded as they reported having followed their last BLS course more than 5 years before taking the questionnaire. Finally, the effect of the COVID-19 pandemic on the intention to perform resuscitation is not known as the survey was conducted prior to this crisis.

### Conclusions

Control beliefs, one of the three dimensions of intention to perform resuscitation, decreased significantly within only 6 months after the last BLS course. Restoring this dimension to its immediate post-BLS course state should be the focus of future research to enhance CPR provision by lay rescuers in cases of OHCA. Far beyond technical issues, this can be achieved through short interventions aimed at building self-confidence and capacity to reinforce the need to act in the case of an emergency.

## Appendix

Multimedia Appendix 1 Original survey questions (in French) and English translation.

Multimedia Appendix 2 Univariate analysis of the three dimensions of the intention to perform resuscitation in first-aid course participants with a cutoff of 1 year since completion of the last course.

Multimedia Appendix 3 Univariate analysis of the three dimensions of the intention to perform resuscitation in first-aid course participants with the cutoff at 2 years since completion of the last course.

## Abbreviations

BLS: basic life support
CPR: cardiopulmonary resuscitation
OHCA: out-of-hospital cardiac arrest
