# Supplementary material for: Evolution of Bystander Intention to Perform Resuscitation Since Last Training: Web-Based Survey
Source: JMIR Form Res. 2020 Nov 30;4(11):e24798. doi: 10.2196/24798 (PMC7735898; doi:10.2196/24798)
Supplement: Multimedia Appendix 2 [file formative_v4i11e24798_app2.pdf]

	Last course followed 1 year before or less (N=113)[a]	Last course followed more than 1 year before (N=91) [a]	*P* value
Attitude	***6.00 (5.69 – 6.30)***	***6.15 (5.82 – 6.48)***	***.50***
Q1 – Thinking that performing resuscitation could save a life	0.78 (0.71 – 0.85)	0.82 (0.74 – 0.90)	.45
Q2 – Knowing the importance of starting a resuscitation before EMS arrival	0.92 (0.87 – 0.98)	0.91 (0.83 – 0.98)	.76
Q3 – Not being afraid of disease transmission	0.46 (0.33 – 0.59)	0.73 (0.62 – 0.84)	.002
Q4 – Not being afraid of hurting the victim by performing CPR	0.91 (0.86 – 0.96)	0.82 (0.74 – 0.90)	.05
Q5 – Not being afraid of worsening the victim's condition	0.72 (0.62 – 0.81)	0.74 (0.65 – 0.83)	.72
Q6 – Not being afraid of legal action	0.73 (0.64 – 0.83)	0.58 (0.45 – 0.71)	.06
Q7 – Being proud of performing resuscitation successfully	0.57 (0.46 – 0.68)	0.62 (0.49 – 0.74)	.60
Q8 – Belief that knowing CPR is important for the society	0.90 (0.85 – 0.96)	0.93 (0.90 – 0.97)	.35
Subjective normative beliefs	***1.74 (1.47 – 2.0)***	***1.51 (1.21 – 1.81)***	***.26***
Q1 – Belief that relatives would be proud if the participant performed resuscitation	0.52 (0.42 – 0.64)	0.60 (0.48 – 0.72)	.38
Q2 – Belief that relatives want the subject to resuscitate them if needed	0.51 (0.39 – 0.63)	0.47 (0.34 – 0.60)	.65
Q3 – Knowing that relatives are the most likely victim	-0.03 (-0.16 – 0.11)	-0.02 (-0.17 – 0.12)	.96
Q4 – Diffusion of responsibility	0.73 (0.64 – 0.82)	0.46 (0.32 – 0.61)	.001
Control beliefs	***3.36 (3.07 – 3.66)***	***2.05 (1.64 – 2.46)***	***< .001***
Q1 – Knowledge of the emergency number	0.86 (0.78 – 0.93)	0.70 (0.57 – 0.82)	.02
Q2 – Feeling able to resuscitate	0.49 (0.39 – 0.59)	0.13 (-0.01 – 0.28)	< .001
Q3 – Feeling able to recognize a cardiac arrest	0.63 (0.54 – 0.71)	0.22 (0.09 – 0.35)	< .001
Q4 – Not believing that only health care professionals can adequately perform resuscitation	0.76 (0.67 – 0.84)	0.73 (0.63 – 0.82)	.63
Q5 – Knowing how to perform a resuscitation	0.63 (0.54 – 0.72)	0.27 (0.15 – 0.40)	< .001

[a] Data are presented as mean (95% CI).

For individual questions, scores can range from -1.0 to +1.0. A positive score indicates an answer in favor of the intention to perform resuscitation.
