# Supplementary material for: Evolution of Bystander Intention to Perform Resuscitation Since Last Training: Web-Based Survey
Source: JMIR Form Res. 2020 Nov 30;4(11):e24798. doi: 10.2196/24798 (PMC7735898; doi:10.2196/24798)
Supplement: Multimedia Appendix 3 [file formative_v4i11e24798_app3.pdf]

	Last course followed 2 years before or less (N=113)[a]	Last course followed more than 2 years before (N=91) [a]	*P* value
Attitude	***6.06 (5.82 – 6.34)***	***6.01 (5.57 – 6.45)***	***.82***
Q1 – Thinking that performing resuscitation could save a life	0.79 (0.73 – 0.85)	0.82 (0.69 – 0.95)	.70
Q2 – Knowing the importance of starting a resuscitation before EMS arrival	0.90 (0.85 – 0.96)	0.95 (0.90 – 1.00)	.41
Q3 – Not being afraid of disease transmission	0.53 (0.43 – 0.64)	0.77 (0.62 – 0.91)	.03
Q4 – Not being afraid of hurting the victim by performing CPR	0.89 (0.84 – 0.94)	0.78 (0.65 – 0.91)	.06
Q5 – Not being afraid of worsening the victim's condition	0.74 (0.66 – 0.81)	0.70 (0.54 – 0.85)	.63
Q6 – Not being afraid of legal action	0.72 (0.64 – 0.80)	0.45 (0.24 – 0.67)	.006
Q7 – Being proud of performing resuscitation successfully	0.58 (0.49 – 0.67)	0.62 (0.42 – 0.83)	.71
Q8 – Belief that knowing CPR is important for the society	0.91 (0.88 – 0.95)	0.93 (0.87 – 0.98)	.76
Subjective normative beliefs	***1.76 (1.54 – 1.99)***	***1.15 (0.71 – 1.58)***	***.01***
Q1 – Belief that relatives would be proud if the participant performed resuscitation	0.54 (0.45 – 0.63)	0.65 (0.46 – 0.84)	.29
Q2 – Belief that relatives want the subject to resuscitate them if needed	0.55 (0.45 – 0.64)	0.29 (0.81 – 0.50)	.02
Q3 – Knowing that relatives are the most likely victim	-0.01 (-0.12 – 0.10)	-0.09 (-0.30 – 0.13)	.54
Q4 – Diffusion of responsibility	0.69 (0.61 – 0.77)	0.29 (0.05 – 0.54)	< .001
Control beliefs	***3.09 (2.82 – 3.35)***	***1.55 (0.91 – 2.18)***	***< .001***
Q1 – Knowledge of the emergency number	0.83 (0.76 – 0.90)	0.61 (0.39 – 0.83)	.01
Q2 – Feeling able to resuscitate	0.41 (0.31 – 0.50)	0.02 (-0.20 – 0.25)	< .001
Q3 – Feeling able to recognize a cardiac arrest	0.52 (0.43 – 0.60)	0.17 (-0.02 – 0.37)	< .001
Q4 – Not believing that only health care professionals can adequately perform resuscitation	0.76 (0.69 – 0.83)	0.67 (0.50 – 0.84)	.26
Q5 – Knowing how to perform a resuscitation	0.57 (0.49 – 0.65)	0.07 (-0.12 – 0.27)	< .001

[a] Data are presented as mean (95% CI).

For individual questions, scores can range from -1.0 to +1.0. A positive score indicates an answer in favor of the intention to perform resuscitation.
